# Airway opening pressure in an obese ARDS patient: just the start of airway recruitment

**DOI:** 10.1186/s44158-023-00099-2

**Published:** 2023-05-30

**Authors:** Shinshu Katayama, Giorgio Antonio Iotti, Ken Tonai

**Affiliations:** 1 Division of Intensive Care, Department of Anesthesiology and Intensive Care Medicine, Jichi Medical University School of Medicine, 3311-1, Yakushiji, Shimotsuke, Tochigi 329-0498 Japan; 2 Hamilton Medical AG, Bonaduz, Switzerland

To the Editor,

Small airway closure, as may occur in morbidly obese patients and acute respiratory distress syndrome (ARDS) patients, is supposed to completely interrupt the communication between the proximal airway and some alveoli that remain inflated. In this condition, the total positive end-expiratory pressure (PEEP_TOT_) assessed by end-expiratory occlusion has frequently been found to underestimate the real alveolar pressure. Measurement of the airway opening pressure (AOP) on a static pressure–volume (PV) curve obtained by low-flow inflation was proposed as a better alternative, enabling the correct calculation of driving pressure and the convenient setting of PEEP to at least the level of AOP [1].

In this case report, we describe a morbidly obese ARDS patient with an AOP of 12 cmH_2_O as measured by a slow inflation maneuver, but also with airway closure at 16 cmH_2_O as confirmed by respiratory mechanics’ measurements and four-dimensional computed tomography (4D-CT) during dynamic changes of PEEP between 5 and 16 cmH_2_O.

The 51-year old female, morbidly obese patient (body mass index 56.2 kg/m^2^ with predicted body weight 47.9 kg) was intubated for septic shock and ARDS. After stabilization, as we started to de-escalate the ventilatory support, the reduction in PEEP led to a major increase in the patient’s inspiratory effort. A static inflation PV curve, which was obtained using the slow pressure-ramp method at 2 cmH_2_O/s [2] on a HAMILTON-G5 ventilator (Hamilton Medical, Bonaduz, Switzerland), showed an AOP of 12 cmH_2_O (Fig. 1). A 4D-CT at PEEP levels of 5 and 16 cmH_2_O showed no relevant difference in end-expiratory lung volume (EELV) or regions of ventilation between the two levels. There was no difference in peak inspiratory pressure (PIP), plateau pressure (Pplat) or esophageal pressure (Pes) between the two PEEP levels, while PEEP_TOT_ was 15 and 18 cmH_2_O, respectively (Fig. 1 and Table 1).

**Fig. 1:**
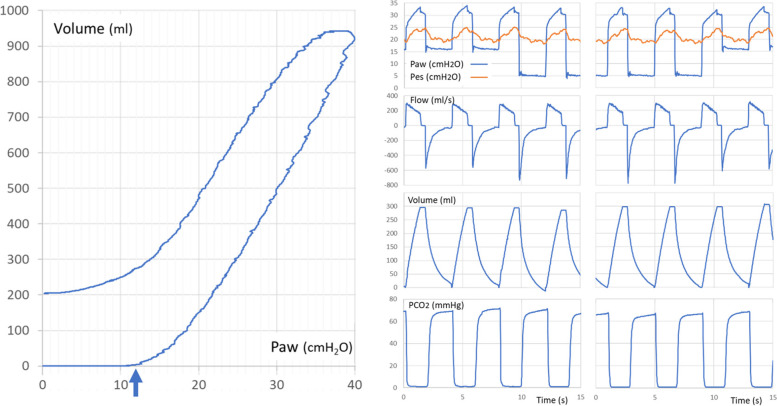
Left panel: Respiratory system static pressure–volume loop by slow inflation-deflation with pressure ramps of 2 cmH_2_O/s. The arrow indicates the airway opening pressure (AOP). Right panel: Airway pressure (Paw), esophageal pressure (Pes), airflow, volume change and capnography during PEEP steps from 16 down to 5 cmH_2_O and back

**Table 1:** Respiratory mechanics data at PEEP 5 and 16 cmH_2_O

	PEEP 5 cmH_2_O	PEEP 16 cmH_2_O
PIP, cmH_2_O	33	33
Pplat, cmH_2_O	29	29
PEEP_TOT_, cmH_2_O	15	18
PEEP_I_, cmH_2_O	10	2
ΔP, cmH_2_O	14	11
V_T_, mL	300	300
Pes,ei, cmH_2_O	25	25
Pes,ee, cmH_2_O	20	20
C_TOT_, mL/cmH_2_O	21	27
Cw, mL/cmH_2_O	60	60
Clung, mL/cmH_2_O	33	50
Lung volume,ee, mL	713	730
Lung volume,ei, mL	995	1011
ΔEELV, mL	–	16

*Abbreviations*: *Clung* lung compliance, *C*_*TOT*_ Respiratory system compliance (total), *Cw* chest wall compliance (chest wall), *EELV* End-expiratory lung volume, *Lung volume, ee* End-expiratory lung volume, *Lung volume, ei* End-inspiratory lung volume, *PEEP*_*I*_ Intrinsic PEEP, *PEEP*_*TOT*_ Total PEEP, *PIP* Peak inspiratory pressure, *Pplat* Plateau pressure, *∆P* Driving pressure, *Pes,ee* End-expiratory esophageal pressure, *Pes,ei* End-inspiratory esophageal pressure, *V*_*T*_ Tidal volume

Gas content volumes were calculated from computed tomography (CT) numbers analyzed from -1000 Hounsfield unit (HU) to 0 HU in each voxel [3]. Lung volume was calculated using the following formula: Gas content volume = SUM (-CT number / 1000 * voxel volume)

## Discussion

In this patient, an AOP of 12 cmH_2_O measured on the static PV curve was lower than the PEEP_TOT_ measured by end-expiratory occlusion. In addition, a step change in PEEP below 16 cmH_2_O was not associated with any relevant change in EELV. Based on these results, we would make the following remarks.

### Unchanged EELV and end-expiratory Pes (Pes,ee) despite dynamic changes in PEEP

A change in EELV must lead to a change in Pes,ee proportional to the chest-wall elastance. In our case, the absence of any change in EELV at PEEP levels between 16 and 5 cmH_2_O as assessed by 4D-CT is consistent with the lack of change observed in Pes,ee and the lack of any relevant temporary imbalance between inspiratory and expiratory tidal volumes (Fig. 1). The static PV curve showed an AOP of 12 cmH_2_O and a volume rise of less than 50 ml at an inflation pressure of 16 cmH_2_O (Fig. 1). We hypothesize that although some airways opened at 12 cmH_2_O, the recruitment of recruitable airways was not complete up to at least 16 cmH_2_O.

### AOP from static inflation PV curves with the slow pressure-ramp method

On a static PV curve obtained using low-flow inflation at 5 l/min, AOP was described as the elastic pressure corresponding to the sharp bend where the actual curve exceeds the PV curve predicted by the ventilator circuit by 4 ml [1]. The slow pressure-ramp method [2] is based on pressure and volume measurements made at the Y-piece of the ventilator circuit. By definition, this kind of measurement excludes any mechanical effect caused by the compliance of the ventilator circuit. Therefore, AOP can easily be identified as the sharp bend where the volume increases and exceeds the baseline by 4 ml, after an initial segment with increasing pressure, but no change in volume (Fig. 1).

### AOP, PEEPTOT and PEEP setting

In our case, AOP was lower than PEEP_TOT_, although it has only been described previously as equal to or higher than PEEP_TOT_ [1]. We found a curvilinear increase in the PV curve slope above the AOP point. This feature of the inflation PV curve may not only depend on different levels of alveolar opening, but also on different levels of airway opening within the inhomogeneous lungs. We hypothesize that the AOP we detected on the static PV curve corresponded to the minimum threshold for communicating with some gas-filled but separated lung areas, while the subsequent curvature was due, at least in part, to progressive communication with the areas requiring more pressure. A PEEP_TOT_ higher than AOP may be indicative of this condition of multiple pressure levels for airway opening that are above the lower one as assessed on the PV curve.

In conclusion, we report the new finding that, at least in morbidly obese patients, PEEP_TOT_ can be higher than AOP. In patients susceptible to small airway closure, both parameters should help us better understand the condition. However, the question remains as to which is the most relevant for calculating driving pressure or making decisions about the PEEP setting. This has never been investigated. The matter may be not so simple, especially if we consider that the recruitment of closed small airways is most likely a phenomenon stratified within a pressure range, rather than limited to a single pressure level.

## Data Availability

The datasets used and/or analyzed during the current study are available from the corresponding author on reasonable request.

## References

[1] Chen L, Del Sorbo L, Grieco DL, Shklar O, Junhasavasdikul D, Telias I (2018). Airway closure in acute respiratory distress syndrome: an underestimated and misinterpreted phenomenon. Am J Respir Crit Care Med.

[2] Nakayama R, Bunya N, Katayama S, Goto Y, Iwamoto Y, Wada K, Ogura K, Yama N, Takatsuka S, Kishimoto M, Takahashi K, Kakizaki R, Sawamoto K, Uemura S, Harada K, Narimatsu E (2022). Correlation between the hysteresis of the pressure–volume curve and the recruitment-to-inflation ratio in patients with coronavirus disease 2019. Ann Intensive Care.

[3] Gattinoni L, Caironi P, Pelosi P, Goodman LR (2001). What has computed tomography taught us about the acute respiratory distress syndrome?. Am J Respir Crit Care Med.

